# Construction and protective efficacy of a novel *Streptococcus pneumoniae* fusion protein vaccine NanAT1-TufT1-PlyD4

**DOI:** 10.3389/fimmu.2022.1043293

**Published:** 2022-10-31

**Authors:** Yali Cui, Chenglin Miao, Wen Chen, Wenling Shang, Qianqian Qi, Wei Zhou, Xia Wang, Yingying Li, Ziyi Yan, Yongmei Jiang

**Affiliations:** ^1^ Department of Laboratory Medicine, West China Second University Hospital, Sichuan University, Chengdu, China; ^2^ Department of Laboratory Medicine, Meishan Women and Children’s Hospital, Alliance Hospital of West China Second University Hospital, Sichuan University, Meishan, China; ^3^ Department of Pediatrics, West China Second University Hospital, Sichuan University, Chengdu, China; ^4^ Key Laboratory of Birth Defects and Related Diseases of Women and Children (Sichuan University), Ministry of Education, Chengdu, China

**Keywords:** *Streptococcus pneumoniae*, fusion protein vaccine, neuraminidase A, elongation factor Tu, pneumolysin, bioinformatics analysis

## Abstract

During the past decades, with the implementation of pneumococcal polysaccharide vaccine (PPV) and pneumococcal conjugate vaccines (PCVs), a dramatic reduction in vaccine type diseases and transmissions has occurred. However, it is necessary to develop a less expensive, serotype-independent pneumococcal vaccine due to the emergence of nonvaccine-type pneumococcal diseases and the limited effect of vaccines on colonization. As next-generation vaccines, conserved proteins, such as neuraminidase A (NanA), elongation factor Tu (Tuf), and pneumolysin (Ply), are promising targets against pneumococcal infections. Here, we designed and constructed a novel fusion protein, NanAT1-TufT1-PlyD4, using the structural and functional domains of full-length NanA, Tuf and Ply proteins with suitable linkers based on bioinformatics analysis and molecular cloning technology. Then, we tested whether the protein protected against focal and lethal pneumococcal infections and examined its potential protective mechanisms. The fusion protein NanAT1-TufT1-PlyD4 consists of 627 amino acids, which exhibits a relatively high level of thermostability, high stability, solubility and a high antigenic index without allergenicity. The purified fusion protein was used to subcutaneously immunize C57BL/6 mice, and NanAT1-TufT1-PlyD4 induced a strong and significant humoral immune response. The anti-NanAT1-TufT1-PlyD4 specific IgG antibody assays increased after the first immunization and reached the highest value at the 35th day. The results from *in vitro* experiments showed that anti-NanAT1-TufT1-PlyD4 antisera could inhibit the adhesion of *Streptococcus pneumoniae (S. pneumoniae)* to A549 cells. In addition, immunization with NanAT1-TufT1-PlyD4 significantly reduced *S. pneumoniae* colonization in the lung and decreased the damage to the lung tissues induced by *S. pneumoniae* infection. After challenge with a lethal dose of serotype 3 (NC_WCSUH32403), a better protection effect was observed with NanAT1-TufT1-PlyD4-immunized mice than with the separate full-length proteins and the adjuvant control; the survival rate was 50%, which met the standard of the marketed vaccine. Moreover, we showed that the humoral immune response and the Th1, Th2 and Th17-cellular immune pathways are involved in the immune protection of NanAT1-TufT1-PlyD4 to the host. Collectively, our results support that the novel fusion protein NanAT1-TufT1-PlyD4 exhibits extensive immune stimulation and is effective against pneumococcal challenges, and these properties are partially attributed to humoral and cellular-mediated immune responses.

## Introduction

Lower respiratory tract infections (LRTIs) are the leading cause of death related to infectious diseases worldwide, with *Streptococcus pneumoniae* (*S. pneumoniae*) being the most important pathogen (1–3). Furthermore, the World Health Organization (WHO) estimates that 14.5 million people have severe pneumococcal disease (PD) and 1.6 million people have died yearly in the early 21st century; thus, PD is causing a growing threat to public health (4, 5). In the history of human struggles against *S. pneumoniae* infection, antibiotics and vaccines have provided acute defenses. The resistance of *S. pneumoniae* to antibiotics, such as penicillins, macrolides, cephalosporins, trimethoprim-sulfamethoxazole (TMP-SMX) or fluoroquinolones, is a global, severe and rapidly developing problem. In particular, penicillin-nonsusceptibility and the resulting cross- or multiresistance are important issues worthy of attention, and these issues complicate treatment decisions, lead to treatment failures and increase the costs of medical care (6). The severe pneumonia epidemic has spread worldwide and become a global fight against epidemics. While the 20th century could be considered a time of antibiotic treatments, the 21st century is a time of vaccine preventions; vaccines are undoubtedly the first choice for preventing pneumococcal infections (7–9).

Since the first commercial usage of pneumococcal vaccines in the 1980s, the incidence of pneumonia has decreased significantly (10). With the ongoing introduction of multivalent PCVs, vaccination and secondary herd protection of nonvaccinated populations has become a public health benefit (11, 12). Since current vaccines are based on capsular polysaccharides and confer only a limited protection against the serotypes included in the vaccine, increased infection with nonvaccine-covered serotypes occurs, which is due to the induction of serotype replacement by vaccine selection pressure (13, 14). It may be impossible to blindly increase serotypes in polysaccharide vaccines due to complex processes and expenses; therefore, developing a better pneumococcal vaccine that targets additional disease-causing serotypes is essential for improving public health through both direct and indirect vaccination effects (12, 15, 16).

Most protein vaccines are highly conserved and can provide serotype-independent protection, as these vaccines are highly immunogenic and can stimulate T cells to produce immune memory; in addition, these vaccines can be mass produced through a simple and low-cost process, which is suitable for use in developing countries (17–19). However, it is difficult for a single protein target to stimulate the host and establish a stable immune response, and too many protein targets affect the specific recognition of the host, resulting in a lower level of antibodies. Recently, much attention has been placed on fusion protein vaccines, as an increasing number of these vaccines have been constructed; these vaccines are developed with recombinant vaccine technology, are based on short immunogenic sequences (20, 21), can effectively stimulate humoral and cell-mediated immune responses instead of large proteins or the whole genome, and do not induce antigenic loads or allergenic responses in the host.

Neuraminidase A (NanA) is well known as a key factor of *S. pneumoniae* in toxicity and adhesion, as NanA cleaves terminal sialic acid residues from oligosaccharide receptors in host epithelial cells to facilitate colonization (22). Elongation factor Tu (Tuf) mediates the binding of aminoacyl-tRNA to ribosome A to promote protein synthesis in bacteria and helps the pathogen escape complement (23). As a powerful virulence factor of *S. pneumoniae*, pneumolysin (Ply), which is a lytic cytosolic protein of *S. pneumoniae*, can not only directly destroy host cells but also has an important impact on the immune response of the host (24). All of these proteins are important virulence proteins that have exhibited good immunogenicity and protective effects against pneumococcal infections in mouse models and human clinical trials (25). In our previous studies, we successfully confirmed that all three proteins are conserved in clinical pneumococcal strains. These encouraging results prompted us to conduct further research, such as constructing the functional epitopes of these proteins and evaluating the immune responses induced by fusion protein vaccines based on the three proteins (26).

Thus, we continued our efforts of developing an *S. pneumoniae* vaccine. In this study, we successfully designed and constructed a novel fusion protein, NanAT1-TufT1-PlyD4, which consisted of a lectin domain from NanA, a high antigenic index sequence containing complete domain 2 from Tuf, and a D4 domain from Ply, which was achieved through a flexible Linker ([GGGGS]_2_) based on bioinformatic features. We immunized C57BL/6 mice to evaluate the vaccine potential. The cellular and humoral immune responses were studied, and we validated the immune-protective effect of the protein against focal and lethal challenge through *in vivo* and *in vitro* models, providing an experimental basis for novel *S. pneumoniae* protein vaccines.

## Materials and methods

### Screening of amino acid sequences and vaccine epitopes

The online database of UniPort (https://www.uniprot.org/) was used to obtain the amino acid sequences of proteins NanA, Tuf and Ply. Then, the online server signalP-5.0 (http://www.cbs.dtu.dk/services/SignalP-5.0/) was used to analyze the signal peptide; transmembrane domains were analyzed using TMPred (https://bio.tools/TMPred). In addition, epitope prediction was performed using IEDB (http://www.iedb.org/) with default parameters (27).

### Structural prediction and validation of the fusion protein vaccine

The structure of the vaccine candidate was obtained by Alphafold2 (https://cryonet.ai/af2/), and the epitopes considered for vaccine construction were visualized by PyMOL. The tertiary structure was validated using PROVE analysis followed by ERRAT score (28). PROVE validated the structure based on the predicted Z score. Furthermore, the quality of the generated model of the fusion protein was determined by Ramachandran plot analysis using the MolProbity Ramachandran map (29) (http://molprobity.biochem.duke.edu/). VaxiJen v2.0 was used to evaluate the antigenicity of the whole construct with a threshold value of 0.4, which is based on the ability of protein sequences to perform auto-cross covariance (ACC) transformation into identical vectors of principal amino acid features (30). Additionally, the allergenicity of the vaccine was checked by the AllergenFP server (31) (https://ddg-pharmfac.net/AllergenF/).

### Prediction of the physicochemical properties

The ExPASy ProtParam online server (https://web.expasy.org/protparam) was employed to appraise the physicochemical parameters of the fusion protein vaccine, including the molecular weight (MW), theoretical isoelectric point (pI), instability index, aliphatic index, half-life, grand average of hydropathicity (GRAVY) and extinction coefficient (32).

### Mice

Specific pathogen-free C57BL/6 mice that were female and 4- to 6-week-old were purchased from Chengdu Dashuo Experimental Animal Co. and raised in the animal house at the Second West China Hospital of Sichuan University. All experimental procedures were approved by the Medical Ethics Committee of the Second West China Hospital of Sichuan University (No. 2018021).

### Bacteria

The *S. pneumoniae* strain ATCC 49619 (serotype 19F) used in this experiment was purchased from the American Type Culture Collection. *Escherichia coli (E. coli)* DH5α (Invitrogen, CA, USA) was used as the host for plasmid cloning. *E. coli* BL21 (Invitrogen, CA, USA) was used as a protein expression carrier. *E. coli* were cultured in Luria-Bertani supplemented with kanamycin antibiotics (100 μg/ml). The different clinical strains of *S. pneumoniae* (which were separated from those in our laboratory and were from different geographical locations, disease states, or body sites) used are listed in **Table 1** (26). The *S. pneumoniae* strains were cultured on TSA plates supplemented with 5% sheep blood (blood agar) or in THY medium in an atmosphere of 5% carbon dioxide and 95% air at 37°C.

**Table 1:** Clinical strains of *Streptococcus pneumoniae* in the experiments.

Serial number	Strain	Serotype	Sequence type (ST)	PMEN
1	ZMU3H13628	19F	ST271	Taiwan^19F^-14
2	WCSUH32904	19A	ST 320	N/A
3	WCSUH31937	6A	ST 473	N/A
4	JJMCH670811	6B	ST 902	N/A
5	ZMU3H17501	14	ST 876	N/A
6	WCS20276	23F	ST 81	N/A
7	ZMU3H14496	34	ST 4640	N/A
8	WCSUH32403	3	ST 4655	N/A
9	JJMCH597768	23A	ST 16329	N/A
10	WCSUH30174	16F	ST 6542	N/A

The accession numbers of these strains are PRJNA681770, PRJNA656156 and PRJNA643306 at https://www.ncbi.nlm.nih.gov/genbank/. N/A, Not applicable.

### Construction and validation of the fusion protein NanAT1-TufT1-PlyD4

Beijing Tsingke Biotechnology Co. (Ltd.) was entrusted to synthesize the target gene fragment, and the gene sequence was referred to as *S. pneumoniae* standard strain R6 (NC_003098.1). The recombinant expression pET28a plasmid that contained the in frame of the *nanT1-tufT1-plyD4* DNA construct was identified by Sanger sequencing after transforming the *E. coli* DH5α competent strain. The target protein was expressed in *E. coli* BL21(DE3). Liquid cultures were induced overnight at 18°C with 0.4 mM isopropyl β-D-thiogalactoside (IPTG), lysed with Bugbuster HT (Novagen), and subsequently purified over a His-Select Ni^2+^-NTA gravity column (Cytiva, USA), anion exchange chromatography column (Cytiva, USA), gel filtration chromatography column (Cytiva, USA), and polymyxin B resin gravity column (GenScript, USA).

The purity of the fusion protein was evaluated by SDA-PAGE, while the concentration was determined by the bicinchoninic acid (BCA) method. For Western blot analysis, the purified fusion protein was subjected to 10% SDS−PAGE, electrophoretically transferred to polyvinylidene difluoride membranes (Beyotime Biotechnology, Shanghai, China) and detected by an indirect antibody immunoassay.

### Mouse immunization

Six- to eight-week-old female C57BL/6 mice were administered subcutaneously with a 200 μl mixture comprising 20 μg fusion protein or three full-length proteins that were all emulsified with aluminum hydroxide adjuvant (v:v = 1:1) on day 0, and subsequent booster injections were conducted with the same components on days 14 and 28. All the control mice received PBS with adjuvant in the same process or PPV23 (100 μl/per mouse) on day 0 (33).

### ELISA analysis of specific antibody and IgG subtypes

On the 7th, 14th, 21st, 28th and 35th days after the first immunization, the anti-NanAT1-TufT1-PlyD4 antisera were collected from the immunized mice. The fusion protein NanAT1-TufT1-PlyD4 was diluted with an antigen coating solution to 5 μg/mL, 100 μl/well was added to a 96-well plate, and the plate was incubated at 4°C overnight. After washing and blocking were performed, the titers of specific IgG were determined by enzyme-linked immunosorbent assay (ELISA) *via* FITC-labeled goat anti-mouse IgG (Southern Biotech, USA). In addition, IgG subtypes were detected using fluorescein isothiocyanate (FITC)-conjugated goat anti-mouse IgG1, IgG2a, IgG2b and IgG3 (Southern Biotech, USA) following the manufacturer’s instructions as specific IgG. We expressed the antibody titers as the reciprocal of the highest dilution with an absorbance 2.1-fold higher than the background absorbance.

### Western blot analysis of the reactivity with antisera against different strains of *S. pneumoniae*

We selected the top 10 prevalent serotypes of the clinical strains separated from our laboratory, namely, 19F, 19A, 6A, 6B, 14, 23F, 34, 3, 23A and 16F. Whole-cell lysates prepared from the above strains were quantified by the BCA total protein concentration assay (Beyotime Biotechnology, Shanghai, China), subjected to 10% SDS−PAGE and transferred electrophoretically to polyvinylidene difluoride membranes (Bio-Rad, CA, USA). Each blot was reacted with antiserum (1:1000 dilution) from mice immunized with the fusion protein NanAT1-TufT1-PlyD4, and then indirect antibody immunoassay was used to test reactivity with antiserum against different clinical strains with horseradish peroxidase-labeled anti-mouse IgG (Southern Biotech, AL, USA) diluted 1:1000. The reaction was tested with a Pro-light chemiluminescence kit (Tiangen Biotech, Beijing, China).

### Immunofluorescence validation of the anti-NanAT1-TufT1-PlyD4 antiserum reaction with *S. pneumoniae*

A549 cells (Type II epithelial lung carcinoma cells, ATCC) were cultured on 24-well plates with Dulbecco’s modified Eagle’s medium (DMEM) accompanied by 10% fetal bovine serum (FBS) without antibiotics to a concentration of 3×10^5^ cells/ml. *S. pneumoniae* (19F, ZMU3H13628) (5×10^6^ CFU/ml, 0.1 ml) was incubated with the anti-NanAT1-Tuf-PlyD4 antisera for 30 mins. Then, the bacterial suspension was added into the A549 cell culture wells and incubated for 1 h at 37°C. Next, FITC-labeled goat anti-mouse IgG (Beyotime Biotechnology, Shanghai, China) was added to indicate the reacted cells, which were photographed and merged. The antisera from adjuvant-immunized mice were used as controls.

### Inhibition of *S. pneumoniae* adhesion to A549 cells

For the adhesion of the specific anti-NanAT1-TufT1-PlyD4 antiserum, the wells were washed, and A549 cells were digested with 0.25% trypsin-ethylenediamine tetraacetic acid (EDTA) for 5 min at 37°C. After that, the suspension was serially diluted, plated onto TSA plates and cultured overnight. All adhesion experiments were carried out in triplicate. Colony-forming units (CFU) of different groups on the plates were calculated, and then the antiserum that was specific to fusion proteins was evaluated regarding its capacity to inhibit *S. pneumoniae* (19F) adhesion to A549 cells.

### Protection against colonization in a mouse model

On the 14th day of the last immunization, mice in each group (n=8) were intraperitoneally anesthetized with 1.5% sodium pentobarbital solution in advance and then intranasally challenged with *S. pneumoniae* (19F) bacterial suspension (5.0×10^7^ CFU/30 μl). Three days after the intranasal challenge, the whole lung lobes of mice were removed to prepare lung tissue homogenate. All collected samples were serially diluted and plated on Columbia blood plates, and then CFUs on the plates were calculated after an overnight incubation was performed at 37°C in 5% CO_2_. In addition, the intact lung lobes of *S. pneumoniae*-infected mice in the immunized group NanAT1-TufT1-PlyD4 and PBS in the control group were removed to prepare tissue sections and stained with hematoxylin and eosin (H&E) for microscopic examination.

### Lethal challenge in mouse models

Mice were intraperitoneally injected with serotype 3 (NC_WCSUH32403, 1×10^4^ CFU/30 μl) to establish the *S. pneumoniae* lethal infection model on day 14 after the last immunization. The state of the mice was observed at a fixed point for 21 consecutive days. Then, the survival curves of the mice in each group (n=12) were generated, and Cox regression analysis was used to compare whether there were significant differences.

### Determination of cytokines with flow cytometry

The spleens were removed from immunized mice 7 days after the last immunization. The splenocytes were then washed and resuspended in RPMI 1640 (Solarbio, Beijing, China). The cells were cultured in 24-well plates and then stimulated with the fusion protein NanAT1-TufT1-PlyD4 (5 μg/ml) at 0 h. Then, the Th1-, Th2- and Th17-related cytokines IL-2, IL-8, IL-10, IL-17A, IFN-γ and TNF-α were monitored for 72 h at 37°C in 5% CO_2_. The cytokines in culture supernatants were measured by Cytometric Bead Array (CBA, BD Biosciences, USA) *via* flow cytometry.

### Statistical analysis

Statistical Package for Social Science (SPSS) software for Windows was used to assess the statistical significance of the data (version 22.0; Chicago, IL, USA). The chi-square test, Fisher’s exact test, T test, one-way ANOVA statistical analysis and Mantel−Cox test were used. On the basis of the chi-square test, the Bonferroni method was used to test whether the differences among multiple groups were statistically significant; on the basis of the one-way ANOVA statistical analysis, the LSD method and Tamhane’s T2 method were used to test whether the differences among multiple groups were statistically significant. *P* values < 0.05 were considered statistically significant.

## Results

### The design of the fusion protein vaccine NanAT1-TufT1-PlyD4

**Figure 1:**
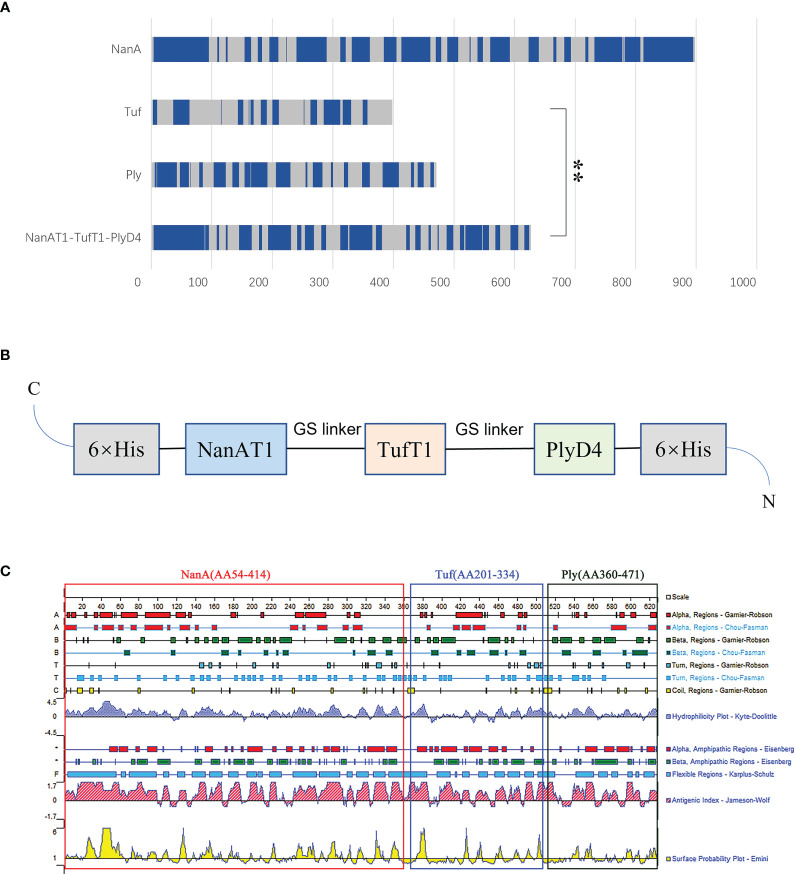
Design of the fusion protein vaccine NanAT1-TufT1-PlyD4. NanAT1, TufT1, and PlyD4 were selected to construct the fusion protein NanAT1-TufT1-PlyD4 by bioinformatics analysis. **(A)** The B-cell epitope prediction results. Bepipred 2.0 from the IEDB server was used for predicting linear B-cell epitopes with default parameters. The residues with scores above the threshold (default value is 0.5) were predicted to be part of an epitope and are colored blue on the graph. “**” indicates the predicted number of B-cell epitopes for the two groups with significant differences, and *P*<0.001. **(B)** Domain pattern of the fusion protein NanAT1-TufT1-PlyD4. The (GGGGS)_2_ linker was designed to connect domains, the pET-28a expression vector was used, and 6×His Tag was added for protein expression and purification. **(C)** Primary sequence analysis of the fusion proteins NanAT1-TufT1-PlyD4. The secondary bioinformatics analysis of the sequence was carried out by DNAstar, and the Jameson-Wolf method suggested that the fusion protein exhibited a relatively high antigenic index.

In this study, the antigenic epitope sequences in the amino acid sequences of the proteins NanA, Tuf and Ply were screened by examining comprehensive gene conservation, protein structure localization, biological functions and related literature reports. The signal peptide sequence of the protein was removed, and the extracellular domain and hydrophilic region were selected. Then, the linear B-cell epitope, hydrophilicity, flexibility, accessibility, turns, exposed surface and polarity of proteins were analyzed by the online software IEDB. Based on the predicted results of binding to B-cell epitopes (**Figure 1A**), the sequence of the fusion protein NanAT1-TufT1-PlyD4 was constructed. The NanAT1-TufT1-PlyD4 fusion protein (**Figure 1B**) consists of 627 amino acids. The lectin-binding domain NanAT1 was chosen (AA54-414 from NanA); the high antigenic index sequence TufT1 was chosen (AA201-334 that contains domain 2 from Tuf); and the domain 4 PlyD4 (AA360-471 from Ply) was chosen. The sequence of the fusion protein NanAT1-TufT1-PlyD4 was secondarily analyzed by a bioinformatics method (**Figure 1C**).

### Structural prediction and validation of NanAT1-TufT1-PlyD4

**Figure 2:**
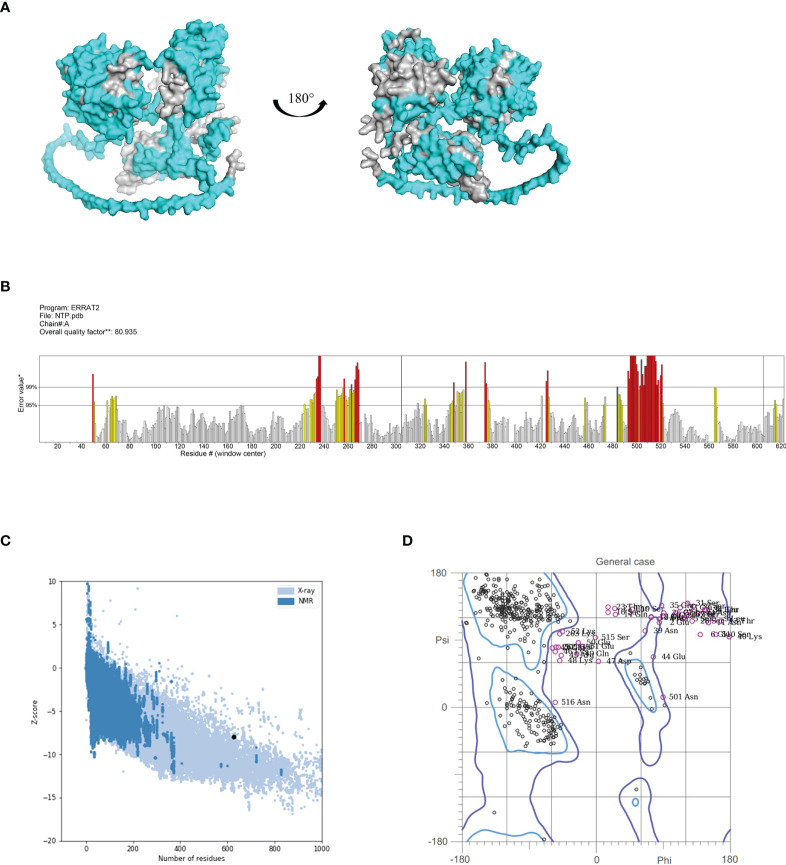
**(A)** 3D model of the fusion protein vaccine construct. Amino acid residues with scores above the threshold (default 0.5) are shown in cyan, and those below the threshold are shown in gray. **(B)** ERRAT plot for the construct with a score of 80.93. Red indicates the misfolded region, yellow identifies the error region between 95% and 99%, and white shows the region with a lower error rate. **(C)** Vaccine 3D structure validation with a Z score of -7.94 using ProSA, the model that lies inside the score range. **(D)** The Ramachandran plot using MolProbity demonstrated that 85.3%, 5.9%, and 8.8% of the residues were in the favored, allowed and outlier regions, respectively.

The 3D structure of the fusion protein vaccine NanAT1-TufT1-PlyD4 was predicted using Alphafold2, and the epitopes considered for vaccine construction were visualized (**Figure 2A**). To validate the structural quality of the constructed vaccine, the ERRAT score, Z score, and Ramachandran plot were presented. The overall quality factor predicted by ERRAT was 80.93, and because higher scores indicate higher quality, this model was considered a high-quality model as the value > 50 was in the generally accepted range (34) (**Figure 2B**). Moreover, the Z score for NanAT1-TufT1-PlyD4 was -7.94, implying that the construct was reliable (35, 36) (**Figure 2C**). The predicted structure was evaluated by MolProbity and was used to generate the Ramachandran plot. The Ramachandran plot analysis of the 3D structure revealed that 85.3% of the residues were in favored regions, 5.9% of the residues were in allowed regions and 8.8% of the residues were in outlier regions (**Figure 2D**). Typically, more than 85% of residues in favored regions is acceptable (37, 38), which confirms the reliability. Additionally, the antigenicity of NanAT1-TufT1-PlyD4 was estimated at 0.7136 (threshold: 0.4) by VaxiJen v2.0. Using the AllergenFP server, the allergenicity of the fusion protein vaccine NanAT1-TufT1-PlyD4 was assessed, indicating that our construct is nonallergen.

### Prediction of the physicochemical property of NanAT1-TufT1-PlyD4

The physicochemical property parameters associated with NanAT1-TufT1-PlyD4 were predicted using ExPASy. Accordingly, the construct 627-amino acid sequence possessed a molecular weight of 69.54 kDa, and the theoretical pI was 5.78, which indicated a moderately acidic nature. The instability index of NanAT1-TufT1-PlyD4 was 31.82, rendering the fusion protein stable. The aliphatic index of the vaccine was 72.89, which suggested that the vaccine exhibited a thermostable ability. The estimated half-life of the construct was calculated to be 0.8 h in mammalian reticulocytes, 10 min in yeast and 10 h in *E. coli*. The total average of hydropathicity (GRAVY) index was -0.773, which indicated that the fusion protein vaccine is hydrophilic, as the lower the score is, the better the solubility. At the same time, the fusion protein extinction coefficient was 83310 M^-1^cm^-1^ at 280 nm when measured in water.

### Western blot validation of the fusion protein NanAT1-TufT1-PlyD4

**Figure 3:**
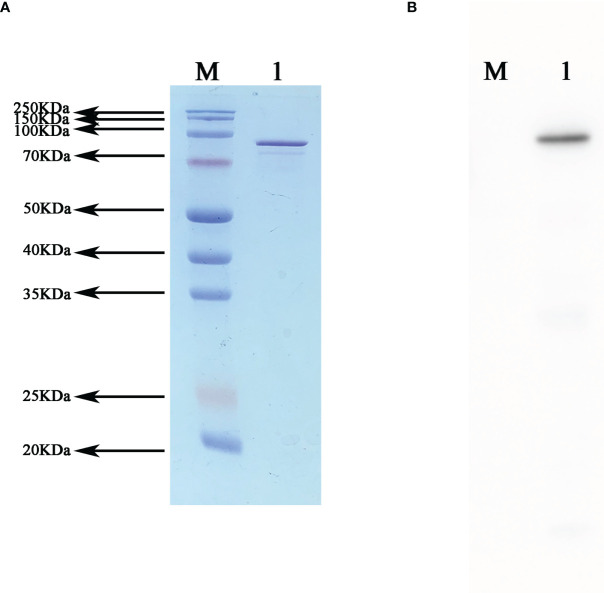
Western blot validation of the recombinant fusion protein NanAT1-TufT1-PlyD4. **(A)** SDS−PAGE of recombinant fusion protein NanAT1-TufT1-PlyD4 was stained with Thomas Brilliant Blue, and the target bands are shown at the corresponding molecular weights. **(B)** Western blot verification was performed after transferring PVDF membranes, and positive bands are shown at the corresponding position.

After purification and SDS PAGE were performed with the expressed fusion protein, clear target bands were obtained at the corresponding position. Western blot analysis was performed using mouse anti-6×His tag monoclonal antibody and goat anti mouse IgG-HRP, and the positive band was visible after exposure (**Figure 3**).

### Preparation of antisera and detection of the specific antibody and IgG subtypes

ELISA analysis of the antisera from groups of mice immunized with the fusion protein NanAT1-TufT1-PlyD4 showed that antigen-specific antibody responses were generated, and the IgG antibody titer gradually increased with the progress of immunization. On the 35th day after the first immunization, the antibody titers of each group were as follows: the NanAT1-TufT1-PlyD4 adjuvant immunization group was (2.99 ± 1.75) × 10^6^ (n=6); the NanAT1-TufT1-PlyD4 without adjuvant immunization group was (8.53 ± 3.30) × 10^4^ (n=6); and the aluminum hydroxide adjuvant control group and PBS control group antibody potency was <1.00 × 10^1^.

In the adjuvant immunization group of fusion protein NanAT1-TufT1-PlyD4, the specific IgG antibody assay was significantly higher than that of the corresponding unadjuvanted group (*P*=0.004), and the antibody titers in the negative control group were less than 1:100; in addition, in the adjuvant immunization group, the specific IgG antibody assay was >1.00×10^5^, which satisfied the requirements of the subsequent experiments.

**Figure 4:**
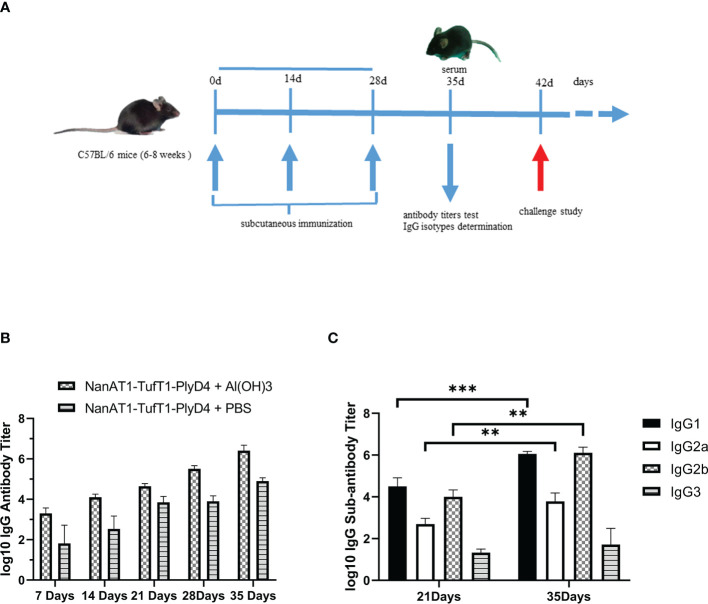
Changes in serum-specific IgG antibodies and subtypes of mice immunized with NanAT1-TufT1-PlyD4. **(A)** Timeline for mouse immunization. Six- to eight-week-old female C57BL/6 mice were immunized subcutaneously on days 0, 14, and 28 of the immunization schedule. On the 35th day, antibody titer tests and IgG subtype determinations were performed, and antisera were prepared. The challenge study was conducted on day 42. **(B)** Time course of antibody induction in immunized mice. Antibody titers in the negative control group were < 1:100, n=6. **(C)** Levels of IgG subtypes in mice immunized with the NanAT1-TufT1-PlyD4 + Al(OH)_3_. Data are significantly different: ** *P*<0.01; *** *P*<0.005. n=6.

Furthermore, to reveal the type of immune response, on the 7th, 14th, 21st, 28th and 35th days after the first immunization, IgG1, IgG2a, IgG2b and IgG3 were determined by indirect ELISA (**Figure 4**). The results demonstrated that the levels of IgG1, IgG2a and IgG2b produced in splenocytes were significantly higher with the immune boost.

### Western blot analysis of reactivity with antisera against different strains of *S. pneumoniae*

**Figure 5:**
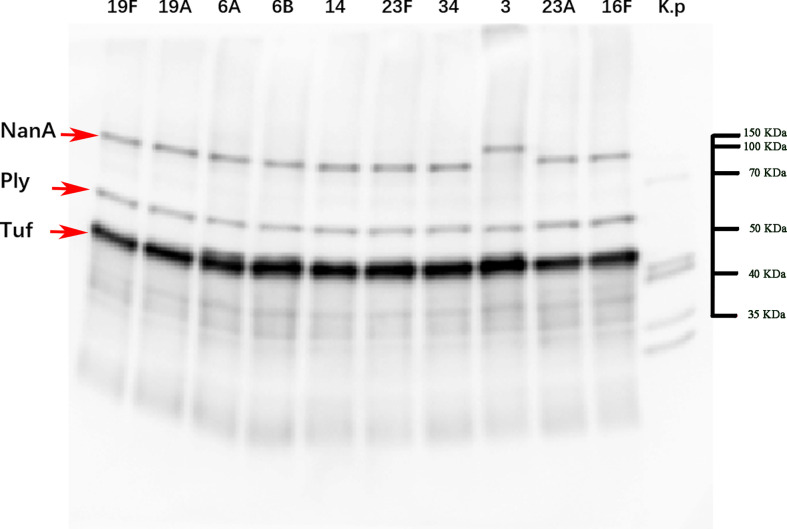
Western blot imaging results of strains with a broad spectrum of anti-NanAT1-TufT1-PlyD4 antiserum. Line 1-10 use the bacterial lysate of the top ten serotypes in the region as the antigen, and Line *K.p* uses the standard strain (ATCC 4352) of *Klebsiella pneumoniae* as the control; “→” indicates the target positive band. According to the results of Coomassie brilliant blue staining, markers of corresponding molecular weights were marked on the original figure.

Cross-immunization was carried out with a variety of serotype strains as antigens to verify the broad spectrum of the anti-NanAT1-TufT1-PlyD4 antisera. Positive bands for NanA (99.74 kDa), Tuf (43.97 kDa) and Ply (52.92 kDa) were observed at the corresponding molecular weights (**Figure 5**).

### Immunofluorescence validation of the anti-NanAT1-TufT1-PlyD4 antiserum reaction with *S. pneumoniae*

**Figure 6:**
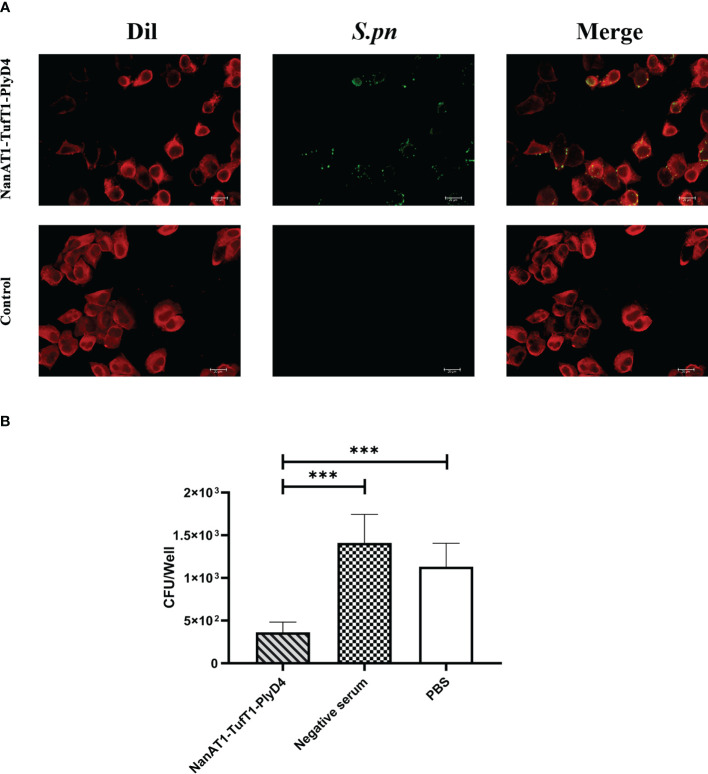
Adhesion of *S. pneumoniae* to A549 cells and inhibition of anti-NanAT1-TufT1-PlyD4 antisera. **(A)** Immunofluorescence imaging results of *S. pneumoniae*, and bacterial adherence to A549 cells. Dil: Dil probe that labels A549 cell membrane; red fluorescence indicates A549 cells. *S. pneumoniae*: NanAT1-TufT1-PlyD4 antisera reacted with intact *S. pneumoniae*, and FITC-goat anti-mouse IgG was then added; Green fluorescence indicates *S. pneumoniae* bacteria. Merge: a composite image of imaging results at different wavelengths, indicating that *S. pneumoniae* bacteria adhere to A549 cells. **(B)** The effect of anti-NanAT1-TufT1-PlyD4 antisera on the number of *S. pneumoniae* adherent A549 cells. A lower colony-forming unit value indicates a stronger inhibitory ability of the serum. (*** *P*<0.005).

The A549 cell line was used as the *in vitro* adhesion carrier, *S. pneumoniae* (serum type 19F, ST271) was used as the antigen, anti-NanAT1-TufT1-PlyD4 antisera were aliquoted for each sample, and FITC-conjugated goat anti-mouse IgG was then added to verify the immune response between anti-NanAT1-TufT1-PlyD4 antisera and intact bacteria. In immunofluorescence imaging, a FITC green fluorescence signal was observed in the experimental group (**Figure 6A**). The experiment not only verified the cross-reactivity between the antiserum and intact *S. pneumoniae* but also constructed a cell adhesion model.

### Evaluation of the inhibition of *S. pneumoniae* adhesion to A549 cells

After *S. pneumoniae* (serotype 19F, ST271) was inhibited by anti-NanAT1-TufT1-PlyD4 antisera, the strains that successfully adhered to A549 cells were counted by the dilution plate coating method. The colony-forming units were as follows (**Figure 6B**): NanAT1-TufT1-PlyD4, (3.6 ± 1.2) × 10^2^ CFU (n=6); mouse negative serum control, (1.4 ± 0.3) × 10^3^ CFU (n=6); and PBS control, (1.1 ± 0.3) × 10^3^ CFU (n=6). Compared with the control group, the *S. pneumoniae* treated with anti-NanAT1-TufT1-PlyD4 antisera showed a significant decrease in the number of cells adhered to A549 cells (*P*<0.001).

### Protection against colonization in a mouse model

**Figure 7:**
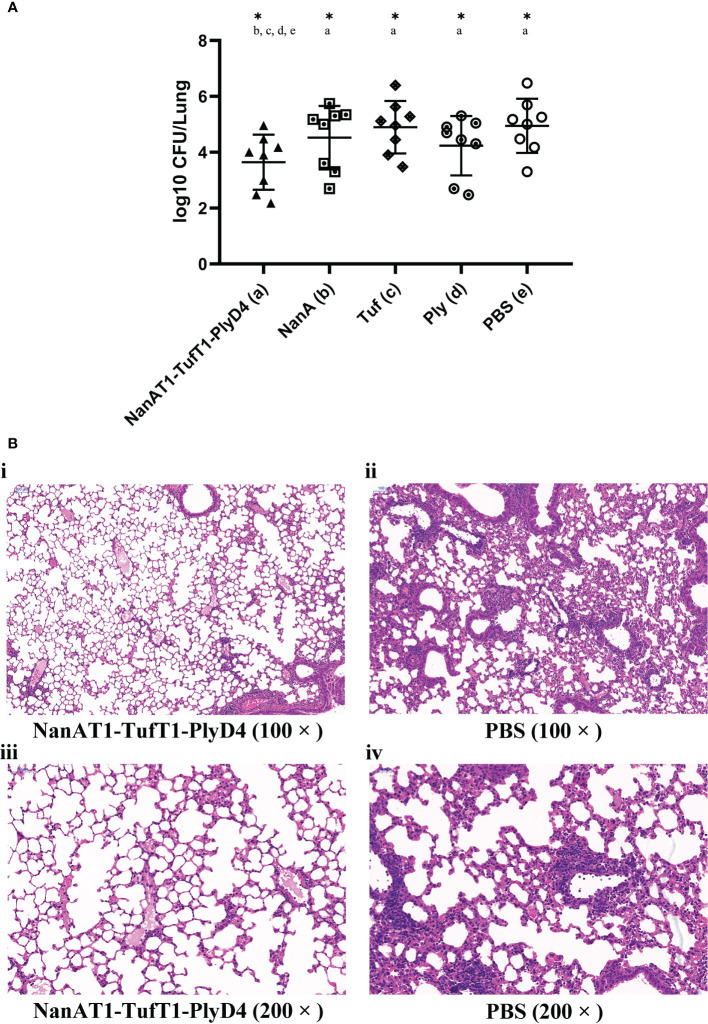
Lung bacterial load and histopathological changes on the 3rd day after intranasal challenge with *S. pneumoniae* in mice. **(A)** The bacterial load in the lungs of mice in the immunized group on the 3rd day after intranasal challenge with *S. pneumoniae*. A lower colony-forming unit value indicates a higher bacterial clearance effect in the lungs. “*” indicates that there is a significant difference between the group and the marked groups (*P*<0.05), n=8. **(B)** Pathological changes in the lung tissue in mice immunized with the fusion protein antigen NanAT1-TufT1-PlyD4 on day 3 after intranasal challenge. Microscopically, inflammatory cells were exuding, capillaries were dilated and congested, and some alveolar spaces were filled with exudate in all mice. Compared with the control group, the inflammatory cell exudation and damage to the alveolar structure in the immunized group was relatively mild, which was consistent with the typical pathological changes in lobar pneumonia caused by *S. pneumoniae*.

Mice were sacrificed on day 3 postchallenge, and lung tissues were isolated to harvest homogenates. After serial dilution, plate counts were performed to determine the CFUs of *S. pneumoniae* colonized in mouse lungs. The statistical results are as follows (**Figure 7**): (a) NanAT1-TufT1-PlyD4: (1.9± 1.0) × 10^4^ CFU (n=8); (b) NanA: (1.5 ± 0.6) × 10^5^ CFU (n=8); (c) Tuf: (4.2 ± 3.0) × 10^5^ CFU (n=8); (d) Ply: (6.1 ± 2.4) ×10^4^ CFU (n=8); (e) PBS: (4.9 ± 3.6) ×10^5^ CFU (n=8).

Compared with the PBS negative control group, the number of colonized colonies in the lungs of the fusion protein NanAT1-TufT1-PlyD4 immunization group was significantly decreased (*P*=0.037). In addition, the bacterial load in the NanAT1-TufT1-PlyD4 group was significantly lower than that in the single full-length protein-immunized groups (b) NanA, (c) Tuf and (d) Ply (*P*ab=0.045; *P*ac=0.042; *P*ad=0.048) (**Figure 7A**).

To study the histological changes in the animals after immunization, lung tissues were collected on the 3rd day after the last immunization and were processed for HE staining to assess the inflammatory response after vaccination. Representative lung lobes from different groups are shown in **Figure 7B**. HE-stained lung sections from PBS exhibited massive inflammatory cell infiltration.

### Protection against lethal challenge in mouse models

**Figure 8:**
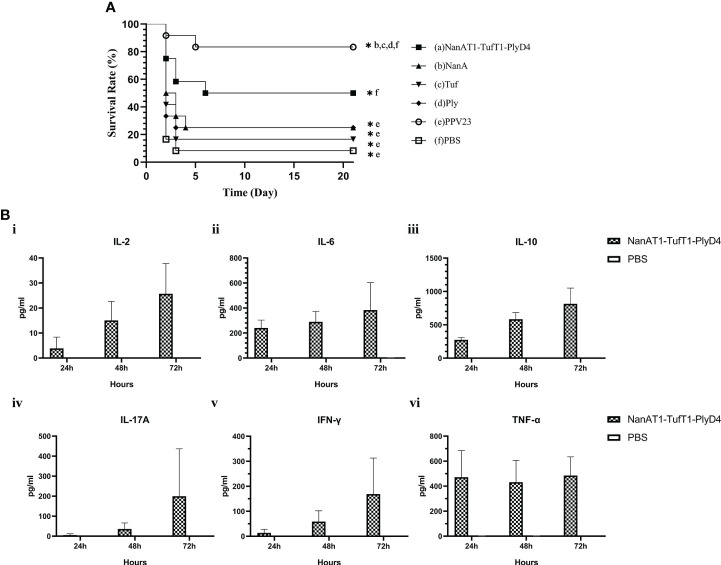
Survival time of the mice and cytokines in splenocytes. **(A)** Analysis of the survival time of mice in the immune group. Mice in each immunization group were observed for 21 consecutive days after the challenge and were tested by the Mantel−Cox model. (a): Fusion protein antigen immunization group; (c–d): single full-length protein immunization group; (e): commercial *S. pneumoniae* polysaccharide vaccine PPV23 immunization group; and (f): PBS/adjuvant control group. Each immunization group n=12. “*” indicates that there is a significant difference between the group and the marked groups (*P*<0.05). **(B)** Changes in cytokine concentrations in the primary culture supernatant of mouse splenocytes after immunization. The levels of IL-2, IL-6, IL-10, IL-17A, IFN-γ and TNF-α produced in splenocytes from intranasally immunized and control mice changed with the culture time; sampling was performed every 24 h (n=3). Data are the means and standard errors of means; “*” represents a significant difference between the 72 h culture and the PBS group.

On the 14th day after the last immunization, mice in each immunized group were intraperitoneally challenged and observed for 21 consecutive days (**Figure 8A**). The 21-day survival rates of mice in each immunization group were as follows: (a) NanAT1-TufT1-PlyD4: 50.0%; (b) NanA: 25.0%; (c) Tuf: 16.7%; (d) Ply: 25.0%; (e) PPV23: 83.3%; and (f) PBS: 8.3%. The survival time of the NanAT1-TufT1-PlyD4 fusion protein immunization group was significantly better than that of the PBS group (*P*=0.007), and there was no significant difference with that of PPV23 (*P*=0.08).

### Cytokine detection by flow cytometry

To further investigate the cytokines involved in the immune responses elicited by intraperitoneal immunization with NanAT1-TufT1-PlyD4, suspensions of splenocytes from the immunized and control groups were grown *in vitro* and stimulated with recombinant NanAT1-TufT1-PlyD4 (5 μg/ml). The results demonstrated that the levels of IL-2 ([25.69 ± 12.06] pg/ml, *P*=0.001), IL-6 ([383.78 ± 219.49] pg/ml, *P*=0.002), IL-10 ([815.40 ± 234.90] pg/ml, *P*<0.001), IL-17A ([200.05 ± 136.80] pg/ml, *P*=0.045), IFN-γ ([168.88) ± 83.02] pg/ml, *P*=0.017) and TNF-α ([485.05 ± 149.07] pg/ml, *P*=0.030) produced in splenocytes from the immunized group were significantly higher than those from the control group (**Figure 8B**). No cytokines (<1.0 pg/ml) were detected in any of the negative control PBS groups.

## Discussion

Despite advances in global vaccine strategies, *S. pneumoniae* remains a disease with a high burden, especially in vulnerable populations, such as young children, adults older than 65 years old, and immunocompromised individuals (3, 39). To date, five licensed pneumococcal vaccines are available to prevent pneumococcal infection. First, Pneumovax 23 (PPV23), which is composed of purified pneumococcal capsular polysaccharide from 23 different serotypes without any adjuvant, was approved for use in persons ≥50 years and ≥2 years who are at increased risk of pneumococcal disease; however, PPV23 exhibits no protective effect on the bacteria colonizing the population, and these bacteria are considered to be reservoirs of drug resistance genes and capsular polysaccharide mutations (17, 40). Second, Prevnar 13 (PCV13), which is composed of 13 serotypes that are individually conjugated to a nontoxic variant of diphtheria toxin protein (CRM_197_), is administered intramuscularly with aluminum phosphate as an adjuvant and exhibits protective effects on vaccine-type (VT) invasive pneumococcal disease (IPD) and community-acquired pneumonia (CAP). Third, PCV15 contains all the antigens in PCV13 and 2 additional antigens, and PCV15 prevents invasive pneumococcal disease caused by the additional 2 non-PCV13 serotypes in adults ≥65 years old and 19-64 years old with chronic medical or immunocompromising conditions (41). Fourth, in June 2021, the FDA-approved Prevnar 20 (PCV20), which is composed of the capsular polysaccharide serotypes contained in PCV13 and 7 additional pneumococcal serotypes that are individually conjugated to CRM_197_, was approved for active immunization of adults ≥18 years old to prevent pneumonia and invasive disease caused by the 20 vaccine serotypes. The additional serotypes can cause IPD and have been associated with high case-fatality rates, antibiotic resistance and meningitis. In addition, there is a novel 24-valent pneumococcal vaccine, ASP3772, which contains 13 serotypes in PCV13, and an additional 11 serotypes have been confirmed to be safe and well tolerated in adults 65 to 85 years of age (41, 42).

The main limitations of commercial vaccines lie in serotype dependence, incomplete protective efficacy and poor memory (43). To date, more than 100 distinct capsular serotypes have been identified, and due to the diversity in pneumococcal capsular types and the constant appearance of serotype replacements, obtaining a universal vaccine for *S. pneumoniae* has become a public health goal to improve the serotype restriction of the current vaccines (17, 44, 45). Ideal candidates for potential vaccines are proteins, such as pneumococcal surface protein A (PspA), pneumococcal surface adhesin A (PsaA), Ply and NanA, that have a high sequence conservation and participate in the biological process of bacterial adhesion and invasion; in addition, these proteins should stimulate immune responses, which can also avoid the problems associated with poor polysaccharide immunogenicity (17, 20). In recent years, molecular technology has significantly developed, and methods to design novel fusion protein vaccines that are composed of several single protein peptides based on bioinformatics have become a new approach to research vaccines. Fusion proteins deserve to be further developed to obtain comprehensive protection with the advantages of promising immunogenicity and conservative, simple production, facilitation of purification, easier standardization and irrespective of prevailing serotypes (45–47).

Reports have shown that immunization with NanA, Tuf, and Ply induced protective effects against focal and lethal infections with different *S. pneumoniae* serotypes, which have been targeted by various scientists to develop a potential vaccine (48–50). Bioinformatic analysis, as one of important and prominent computational tools, could offer deep mechanistic insights into the complexity of biological systems and obtain high-performance candidate vaccines. Based on this, we designed a novel fusion protein combined with the lectin domain from NanA (NanAT1: AA54-414); fragment including domain 2 from Tuf (TufT1: AA201-334), which exhibits a high antigenic index sequence; and the domain 4 from Ply (PlyD4: AA360-471) to construct NanAT1-TufT1-PlyD4.

A key characteristic for an ideal candidate vaccine is the ability to elicit an immune response. IgG can not only directly neutralize toxins but also activates the complement pathway, mediate antibody-dependent cellular cytotoxicity (ADCC), and participates in opsonophagocytosis for immune protection (51). Aluminum hydroxide is a potent enhancer of antibody production and is considered an effective adjuvant; in addition, aluminum hydroxide preferentially primes Th2-type immune responses and has the potential to augment the humoral and cellular immune response when immunized with the fusion protein (52, 53). In our study, the purified fusion protein NanAT1-TufT1-PlyD4 was used to subcutaneously immunize C57BL/6 mice with or without additional alum adjuvant. We measured antigen-specific antibody responses on the 7th, 14th, 21st, 28th and 35th days after the first immunization by indirect ELISA and found that immunization with NanAT1-TufT1-PlyD4 could induce specific IgG antibodies in the sera with no obvious side effects. Since wild-type Ply could interact with Toll-like receptor 4 (TLR4) and induce the activation of the NLR family pyrin domain that contained 3 (NLRP3) inflammasome, wild-type Ply has potential as a mucosal adjuvant for use in combination with other proteins (20). Here, the fusion protein with alum adjuvant exhibited some advantages, including higher IgG titers and increased induction of immune responses; furthermore, with the fusion protein, it is easy to deliver antigens by targeting TLRs on immune cells. Thus, immune adjuvant is still indispensable in the type of fusion protein antigen injection. In addition, we observed a significant difference in the IgG titers in the sera between the first and the last booster. The specific IgG antibody titer could be significantly increased, and the antibody titer was >2×10^6^ on the 7th day after the last immunization, indicating that the protocol of three immunizations was indeed applicable to the fusion protein NanAT1-TufT1-PlyD4, which was also a common immunization protocol for other protein vaccines (54). Additionally, the antibody subtype analysis revealed that IgG1, IgG2a and IgG2b were produced by the immunized group, while a scarce IgG3 response was observed, suggesting that in addition to B-cell immune responses, the immune process also stimulated cellular immune responses of Th1 and Th2, which was similar to other protein vaccine studies (20, 55). All the results provided key insights that the NanAT1-TufT1-PlyD4 fusion protein with alum adjuvant was sufficient to elicit immune and protective responses.

Due to the genetic diversity of the pathogenic strains, a broad-spectrum assessment against heterologous strains is required for protective efficacy assessment. In this study, we found that the anti-NanAT1-TufT1-PlyD4 antisera were cross-reacted with the lysed and denatured proteins from 10 different pathogenic strains of *S. pneumoniae*, suggesting that the antisera of NanAT1-TufT1-PlyD4 could identify the native NanA, Tuf and Ply proteins; furthermore, these results indicated that the antisera potentially exhibited universal protective efficacy.

To discover the function of the anti-NanAT1-TufT1-PlyD4 specific antisera based on their surface recognition of *S. pneumoniae*, cell adhesion assays were carried out *in vitro*, and the results demonstrated that anti-NanAT1-TufT1-PlyD4 antisera inhibited the adhesion of pneumococcus to A549 cells; these results supported that the protection was enhanced through humoral immunity against invasive pneumococcal infection, which was elicited by immunization with NanAT1-TufT1-PlyD4. In addition, we successfully constructed an *S. pneumoniae* adhesion A549 cell model and carried out an indirect immunofluorescence test to verify that the antisera could interact with intact *S. pneumoniae*. The results indicated that the anti-NanAT1-TufT1-PlyD4 antisera could play a protective role in humoral immunity by inhibiting the adhesion process between bacteria and host cells.

Lung’s infection of *S. pneumoniae*, as the most common site of serious infection, represents the combination of both mucosal (nasopharynx) and systemic (septicaemia) sites, and is responsible for high morbidity and mortality worldwide (56). In this study, we established a mouse pulmonary infection model by nasal challenge with a 19F/ST271 strain suspension, imitating the natural infection pathway of *S. pneumoniae*. The results showed that NanAT1-TufT1-PlyD4 was superior to a single full-length protein in protecting immunized mice against intranasal pneumococcal infection. There was a significant reduction in the number of *S. pneumoniae* in the lungs (CFUs), and a better protective effect was observed than that with the three individual recombinant full-length protein antigens. In addition, we observed pathological changes in the lung tissue of mice on day 3 after intranasal challenge and found that the inflammatory cell exudation and damage to the alveolar structure in the immunized group were relatively mild compared with those in the control group, which supported that NanAT1-TufT1-PlyD4 is a superior candidate vaccine against *S. pneumoniae* infection.

Previous studies have demonstrated the protective effects of NanA, Tuf and Ply against *S. pneumoniae* infections (57, 58). Our study showed that immunization with the fusion protein NanAT1-TufT1-PlyD4 could protect mice from lethal intraperitoneal challenge with *S. pneumoniae* serotype 3. The 21-day survival rate of the immunized group was 50%, which met the standard for the marketed vaccine (50% effective rate) stipulated by the WHO. Although this protective effect was weaker than that of PPV23, based on the broad spectrum of its immune target strains, sufficient immunogenicity, convenience for mass production, and related protein characteristics, this protein also showed considerable potential for development into a mature pneumococcal candidate vaccine in further research.

Upon infection, immune cells launch robust inflammatory and antimicrobial responses by stimulating cytokines and chemokines to promptly defend against bacterial infection. After stimulation with the fusion protein, TNF reached its peak at 24 h, and IL-2, IL-6, IL-10, and IFN-γ gradually increased within 72 h, which suggested that both Th1 and Th2 cellular immune responses played a role in the protective effects observed after subcutaneous immunization with NanAT1-TufT1-PlyD4. Additionally, as a proinflammatory cytokine, IL-17A is the main effector of Th17 cells; IL-17A is also an early triggering factor for T lymphocytes to induce inflammatory responses and can amplify inflammation by promoting the release of proinflammatory cytokines, further promoting the activation of T lymphocytes and stimulating epithelial, endothelial, fibroblast and other cells to produce IL-6, IL-8, GM-CSF and other cytokines (22). According to previous reports, the Th17A-mediated immune response can effectively remove pathogenic bacteria on the surface of host mucosal tissue and can avoid the development of invasive infection due to *S.pn* colonization in the nasopharynx (20, 59). In this study, we confirmed that increases in the level of IL-17A secreted by splenocytes could be induced after stimulation with the fusion protein NanAT1-TufT1-PlyD4, suggesting that the Th17-type cellular immune response was successfully stimulated, which was also considered to be among the effector mechanisms that reduce the colonization of mouse lungs after intranasal challenge. Further studies are needed to illuminate the relationship between cytokines and IgG in vaccination.

Our studies suggest that the NanAT1-TufT1-PlyD4 fusion protein is a promising vaccine strategy that enhances the immune response and stimulates the production of high levels of antibodies against pneumococcal strains. We are interested in this fusion protein due to the simplification of the vaccine production process and the ease of quality control, although its protective efficacy is weaker than that of the PPV23 vaccine. Furthermore, it is an issue worth investigating in terms of its protection against fatal or colonization challenges by different strains of *S. pneumoniae*, particularly serotypes not included in PCV13 or PPV23 but commonly associated with disease, such as serotypes 15A, 15C, 29 (26, 41).

In conclusion, we emphasized that NanAT1-TufT1-PlyD4 was highly immunogenic and broadly conserved, and that anti-NanAT1-TufT1-PlyD4-specific antisera inhibited pneumococcal adhesion to host cells. In addition, we clarified that NanAT1-TufT1-PlyD4 effectively reduced the number of *S. pneumoniae* colonizing the lungs of mice, effectively protected them from the fatal challenge of *S. pneumoniae*, and significantly prolonged the survival time of experimental mice; while further studies showed that this protective efficacy was mediated by humoral and cellular immune responses. All our results indicated that the fusion protein NanAT1-TufT1-PlyD4 was a highly efficacious serotype-independent pneumococcal vaccine.

## Data availability statement

The datasets presented in this study can be found in online repositories. The names of the repositories and accession number(s) are included in the article/supplementary material. Further inquiries can be directed to the corresponding authors.

## Ethics statement

The animal study was reviewed and approved by the Laboratory Animal Management and Ethics Committee of West China Second University Hospital, Sichuan University.

## Author contributions

YC, ZY, and YJ conceived the experiments. CM, WS, QQ, and XW performed bioinformatic analysis, YC, CM, WZ, WC, and YL performed the experiments. YC and CM wrote the paper. ZY and YJ edited the final version. All authors contributed to the article and approved the submitted version.

## Funding

This work was supported by the Science & Technology Department of Sichuan Province (No. 2022YFS0239), the Cadres Healthcare Research Projects in Sichuan Province (No. 2021-1703), the Clinical Scientific Research Projects of Sichuan University (NO.18H0629 and NO. 20H0192), and the Science & Technology Department West China Second University Hospital, Sichuan University (No. KL044, No. KL066). The funders had no role in study design, data collection and analysis and interpretation of the data.

## Acknowledgments

We thank Dr. Dong Deng and Dr. Xiang Wang from West China Second Hospital, Sichuan University for their technical suggestions.

## Conflict of interest

The authors declare that the research was conducted in the absence of any commercial or financial relationships that could be construed as a potential conflict of interest.

## Publisher’s note

All claims expressed in this article are solely those of the authors and do not necessarily represent those of their affiliated organizations, or those of the publisher, the editors and the reviewers. Any product that may be evaluated in this article, or claim that may be made by its manufacturer, is not guaranteed or endorsed by the publisher.
